# Effect of grazers and viruses on bacterial community structure and production in two contrasting trophic lakes

**DOI:** 10.1186/1471-2180-11-88

**Published:** 2011-04-29

**Authors:** Lyria Berdjeb, Thomas Pollet, Isabelle Domaizon, Stéphan Jacquet

**Affiliations:** 1 INRA, UMR CARRTEL, Station d'Hydrobiologie Lacustre, BioFEEL Group, 74203 Thonon-les-Bains cedex, France

**Keywords:** Lakes, microcosm, spring-summer variations, bacterial production, viral production, bacterial community structure, grazers

## Abstract

**Background:**

Over the last 30 years, extensive studies have revealed the crucial roles played by microbes in aquatic ecosystems. It has been shown that bacteria, viruses and protozoan grazers are dominant in terms of abundance and biomass. The frequent interactions between these microbiological compartments are responsible for strong trophic links from dissolved organic matter to higher trophic levels, *via *heterotrophic bacteria, which form the basis for the important biogeochemical roles of microbial food webs in aquatic ecosystems. To gain a better understanding of the interactions between bacteria, viruses and flagellates in lacustrine ecosystems, we investigated the effect of protistan bacterivory on bacterial abundance, production and structure [determined by 16S rRNA PCR-DGGE], and viral abundance and activity of two lakes of contrasting trophic status. Four experiments were conducted in the oligotrophic Lake Annecy and the mesotrophic Lake Bourget over two seasons (early spring *vs. *summer) using a fractionation approach. *In situ *dark *vs. *light incubations were performed to consider the effects of the different treatments in the presence and absence of phototrophic activity.

**Results:**

The presence of grazers (i.e. < 5-μm small eukaryotes) affected viral production positively in all experiments, and the stimulation of viral production (compared to the treatment with no eukaryotic predators) was more variable between lakes than between seasons, with the highest value having been recorded in the mesotrophic lake (+30%). Viral lysis and grazing activities acted additively to sustain high bacterial production in all experiments. Nevertheless, the stimulation of bacterial production was more variable between seasons than between lakes, with the highest values obtained in summer (+33.5% and +37.5% in Lakes Bourget and Annecy, respectively). The presence of both predators (nanoflagellates and viruses) did not seem to have a clear influence upon bacterial community structure according to the four experiments.

**Conclusions:**

Our results highlight the importance of a synergistic effect, i.e. the positive influence of grazers on viral activities in sustaining (directly and indirectly) bacterial production and affecting composition, in both oligotrophic and mesotrophic lakes.

## Background

The heterotrophic bacterial community is the most important biological compartment involved in the transformation and mineralization of the organic matter in aquatic systems. It also constitutes a key source of prey for higher trophic levels, i.e. primarily flagellates, but also ciliates and the metazooplankton [1,2]. Our conceptual understanding of the role of heterotrophic bacteria in pelagic systems and in global biochemical cycles is closely linked to our understanding of how their growth rate, abundance, distribution and diversity are controlled [3-5].

Different biotic and abiotic factors have been identified as players acting on the activity and composition of the bacterial community, and resources (organic matter and nutrients) are considered one of the main factors controlling this community [2,6]. However, the roles of bacterivory and viral lysis are not insignificant, and may also strongly affect bacterial abundance, activity and structure. Both heterotrophic nanoflagellate (HNF) grazing and viral lysis are known to be variable causes of bacterial mortality, and can be responsible for 10 to 60% of daily bacterial loss in lacustrine systems [e.g. [7]]. In addition, both processes can impact the size distribution of bacterial communities through 'size-selective mortality' for flagellates [8,9] and 'host-specificity' for viruses [10]. Moreover, viruses can act indirectly on bacterial structure throughout the release of cell debris during lysis activity (enriching the pool of dissolved and particulate organic matter (DOM and POM) and inorganic nutrients) enhancing *in fine *growth and production of some bacterial groups [11,12]. Indeed, whether cells are grazed or lysed can have different ecological and biogeochemical consequences, as the implications for the matter and energy flow through the microbial web will be very different [13,14]. Typically, high rates of viral cell lysis may generate a recycling of nutrients and organic matter at the base of the food web and therefore, less carbon and nutrients may reach higher trophic levels, a process referred to as the viral shunt [13,14]. In contrast, if bacteria are grazed by flagellates, nutrients and energy can reach higher trophic levels *via *the connection between the microbial loop and the classical food chain [15]. Thus, these processes can significantly influence the production of dissolved organic carbon and the recycling of nutrients [14,16] and can impact/modify not only bacterial diversity [9,17] but also the relationship between diversity and ecosystem functioning [18].

A few studies have investigated the individual effects of flagellates or viruses on bacterial communities in terms of abundance, production and diversity (e.g. [7,10,19,20]). However, their combined effects on bacteria, and the comparison between individual and combined effects are still limited [18,21,22]. According to these studies, both viral lysis and protistan bacterivory may act additively to reduce bacterial production and sustain diversity, which could explain the less pronounced blooming species in heterotrophic bacterioplankton than in phytoplankton [22]. However, the opposite effect has also been reported [23]. Moreover, comparisons of the combined effects of viruses and flagellates on the bacterial community according to the trophic status of aquatic systems are scarce and until now, no information has been made available for lacustrine systems. To the best of our knowledge, Zhang *et al*. [22] are the only authors who have investigated these effects taking into account a trophic range within a coastal ecosystem, and the same trend was highlighted [22]. According to these authors, a shift of predator control mechanisms from flagellates in oligotrophic systems to viruses in eutrophic systems could explain the results.

In this study, we collected samples from two peri-alpine lakes (Annecy and Bourget) with substantial differences in their trophic state (oligo- *vs*. mesotrophic, respectively) and we developed treatments with either individual or combined predators of the bacterial community using a fractionation approach (i.e. a physical separation of virus-bacteria and the small eukaryotes). Our main goal was to examine the separated and combined effect of viruses, grazers and small autotrophs (< 5 μm) on the bacterial abundance, production and structure, and to compare it in different environmental conditions. Since the importance of both predators (flagellates and viruses) as potential controlling forces of the bacterial community may display seasonal variations in these lakes [7,8,24], this study was carried out at two contrasting periods (early-spring *vs*. summer), characterized by substantial differences in both the dynamics and structure of microbial communities and environmental conditions [8,25].

Our main findings are that both viral lysis and flagellated bacterivory act additively to sustain bacterial production, probably through a cascading effect from grazer-mediated resource enrichment, whereas their effects on the bacterial community structure remain more subtle. On the whole, the combined effects of viruses and flagellates showed the same trend in both lakes Annecy and Bourget.

## Results

### Initial conditions

#### In situ characteristics of the study sites

Lake Bourget is an elongated and north-south oriented lake situated in the western edge of the Alps (length 18 km; width 3.5 km; area 44 km^2^; volume 3.5 × 10^9 ^m^3^; altitude 231 m; maximum depth 147 m; mean depth 80 m; residence time 8.5 years). Lake Annecy is located in the eastern part of France, at a distance of approx. 50 km from the former, (length 14.6 km; width 3.2 km; area 28 km^2^; volume 1.2 × 10^9 ^m^3^; altitude 447 m; maximum depth of 65 m; mean depth 41 m; residence time 3.8 years). From the end of March to mid-July (i.e. periods during which experiments were conducted), *in situ *temperatures of the two study sites varied between 6.2°C and 20.4°C, while the dissolved oxygen varied more modestly, between 9.7 and 11.7 mg l^-1 ^(Table 1). Differences in the concentration of nutrients (NO_3_, NH_4 _and Ptot) between Lake Annecy and Lake Bourget were principally recorded during the early spring experiments (LA1 and LB1, respectively), with values twice to three-times higher in Lake Bourget (LB1) than in Lake Annecy (LA1) (Table 1). Chl *a *concentration was relatively low (i.e. < 2.8 μg l^-1^) for the four experiments (LA1, LA2, LB1 and LB2). The abundance of heterotrophic bacteria varied between 1.2 and 3.5 × 10^6 ^cell ml^-1^, viruses between 3.7 and 15 × 10^7 ^virus ml^-1^, heterotrophic nanoflagellates (HNF) between 2.6 and 7.6 × 10^2 ^cell ml^-1^, pigmented nanoflagellates (PNF) between 1.4 and 18 × 10^2 ^cell ml^-1^, and picocyanobacteria between 2 and 15 × 10^4 ^cell ml^-1^. These parameters were significantly different (ANOVA, P < 0.05, n = 12) between the four experiments (LA1, LA2, LB1 and LB2), indicating distinct biological characteristics at initial sampling. Seasonal difference in the picocyanobacterial abundance was monitored (ANOVA, P < 0.05, n = 6) in both lakes (Annecy *vs. *Bourget), with values 1.6- to two-times higher in summer (LA2 and LB2) than in early spring (LA1 and LB1). HNF and PNF abundances were significantly higher in Lake Annecy than in Lake Bourget (ANOVA, P < 0.05, n = 12, Table 1). In contrast, viral abundances were always lower in the oligotrophic Lake Annecy.

**Table 1:** Physicochemical and biological characteristics of the sampling sites (2 m depth)

Parameters		LA1	LA2	LB1	LB2
Sampling date		26/03/2007	10/07/2007	02/04/2007	17/07/2007
					
Temperature	°C	6.2	19.6	7.5	20.4
DO	mg l^-1^	10.5	9.7	11.7	10
TOC	mg l^-1^	1.7	2.2	2.1	2.5
NO_3_	mg l^-1^	0.2	0.1	0.5	0.2
NH_4_	μg l^-1^	2.0	1.0	6.0	4.0
PO_4_	μg l^-1^	2.0	3.0	4.0	2.0
P total	μg l^-1^	7.0	6.0	21.0	6.0
					
Chl*a*	μg l^-1^	0.7	2.7	1.2	0.7
Cyanobacteria	10^4 ^cell ml^-1^	9.0 ± 0.5	15.0 ± 1.1	2.0 ± 0.1	12.0 ± 0.8
Het. Bacteria	10^5 ^cell ml^-1^	24.4 ± 0.3	12.3 ± 0.4	35.0 ± 1.2	25.2 ± 2.0
Viruses	10^7 ^part ml^-1^	3.7 ± 0.1	5.1 ± 0.4	8.3 ± 0.3	15.3 ± 0.7
HNF	10^2 ^cell ml^-1^	7.5 ± 1.3	6.9 ± 0.6	2.6 ± 1.3	3.9 ± 1.5
PNF	10^2 ^cell ml^-1^	4.9 ± 1.3	18.0 ± 3.1	1.4 ± 0.2	2.9 ± 0.5

DO, dissolved oxygen; Chl *a*, Chlorophyll *a*; TOC, total organic carbon; NO_3_, nitrate; NH_4_, ammonium; P total, total phosphorus; Het. Bacteria, heterotrophic bacteria; HNF, heterotrophic nanoflagellates; PNF, pigmented nanoflagellates. LA1, March sampling in Lake Annecy; LA2, July sampling in Lake Annecy; LB1, April sampling in Lake Bourget; LB2, July sampling in Lake Bourget. Values are means ± standard deviation of results from triplicate measurements.

#### Conditions in experimental bottles - Effect of filtration

**Figure 1:**
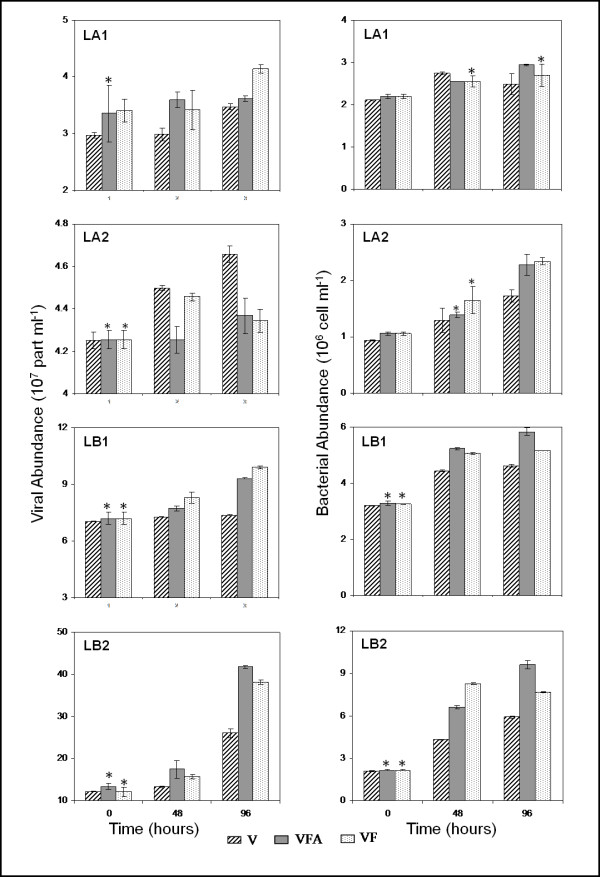
**Time-course of viral abundance (10^7 ^virus ml^-1^) and bacterial abundance (10^6 ^cell ml^-1^) in the four experiments during the incubation period**. Values are given as mean ± standard deviation of triplicate incubations. Asterisks indicate sampling time point for which the VFA and VF treatments were not significantly different from the V treatment (ANOVA, P > 0.05, n = 9). Note that the panels have different scales. LA1, LA2, LB1, LB2: abbreviations as in Table 1.

The < 5-μm prefiltration removed a relatively small fraction of both HNF and PNF (less than 20%), whereas the < 1.6-μm filtration removed, as expected, all of them (Table 2). At the start of the experiments, in VF (Viruses+Bacteria+Flagellates) and VFA (Viruses+Bacteria+Flagellates+Autotrophs) treatments, HNF abundances varied between 2.5 × 10^2 ^cell ml^-1 ^(LB) and 6.5 × 10^2 ^cell ml^-1 ^(LA), PNF between 1.1 × 10^2 ^cell ml^-1 ^(LB) and 14.4 × 10^2 ^cell ml^-1 ^(LA), and picocyanobacteria between 0.7 × 10^4 ^cell ml^-1 ^(LB) and 11.2 × 10^4 ^cell ml^-1 ^(LA) corresponding to 52 to 72% of *in situ *abundances. Comparatively, a small fraction of the picocyanobacterial community passed through the < 1.6-μm filter and only 0.1 and 0.8 × 10^4 ^cell ml^-1 ^were recorded in treatment V (only bacteria and viruses), i.e. 1 to 5% of *in situ *abundance (Tables 1 and 2). In contrast, filtration through 1.6 μm resulted in a small loss of bacterial and viral abundances (less than 14% and 20%, respectively) whereas after 5-μm filtration, loss never exceeded 4% for heterotrophic bacteria and 13% for viruses. At the beginning of the incubation, heterotrophic bacteria and viral abundances, in the four treatments of all experiments varied between 9.4 × 10^5 ^and 33.5 × 10^5 ^cell ml^-1 ^and between 2.9 × 10^7 ^and 13.4 × 10^7 ^virus ml^-1^, respectively (Figure 1). Overall, we succeeded in obtaining incubations with strongly contrasting predator pressure (with or without) and, with negligible loss to the abundances of both bacteria and viruses, when compared to *in situ *conditions.

**Table 2:** Picocyanobacteria (*Synechococcus *spp), pigmented and heterotrophic nanoflagellates (PNF and HNF) abundances at the beginning (t_0_) and at the end (t_final_) of each incubation in the different experiments

Experiments and treatments		Abundance of:					
		
		**Picocyanobacteria (10**^**4 **^**cell ml**^**-1**^**)**		**PNF (10**^**2 **^**cell ml**^**-1**^**)**		**HNF (10**^**2 **^**cell ml**^**-1**^**)**	
		
		**t**_**0**_	**t**_**final**_	**t**_**0**_	**t**_**final**_	**t**_**0**_	**t**_**final**_
LA1							
	V	0.1 ± 0.0	0.3 ± 0.0	0.0 ± 0.0	0.0 ± 0.0	0.0 ± 0.0	0.0 ± 0.0
	VFA	6.5 ± 0.1	7.5 ± 0.1	4.5 ± 1.3	4.8 ± 0.5	6.2 ± 1.3	8.1 ± 1.4
	VF	5.5 ± 0.1	2.4 ± 0.2	4.2 ± 0.2	6.6 ± 0.4	6.5 ± 0.9	8.0 ± 2.6
LA2							
	V	0.8 ± 0.4	0.3 ± 0.2	0.0 ± 0.0	0.0 ± 0.0	0.0 ± 0.0	0.0 ± 0.0
	VFA	10.2 ± 0.1	15.8 ± 0.1	14.4 ± 0.6	28.5 ± 1.3	5.6 ± 0.2	11.1 ± 0.8
	VF	11.2 ± 0.4	6.3 ± 0.3	14.0 ± 0.4	19.1 ± 0.1	5.4 ± 0.3	13.5 ± 0.8
LB1							
	V	0.0 ± 0.0	0.0 ± 0.0	0.0 ± 0.0	0.0 ± 0.0	0.0 ± 0.0	0.0 ± 0.0
	VFA	0.8 ± 0.0	1.5 ± 0.1	1.3 ± 0.5	8.7 ± 0.5	2.5 ± 0.5	12.0 ± 1.7
	VF	0.7 ± 0.2	0.4 ± 0.3	1.1 ± 0.7	6.5 ± 0.2	2.9 ± 0.6	12.4 ± 0.2
LB2							
	V	0.3 ± 0.0	0.5 ± 0.1	0.0 ± 0.0	0.0 ± 0.0	0.0 ± 0.0	0.0 ± 0.0
	VFA	7.3 ± 0.1	16.6 ± 2.1	2.5 ± 2.8	7.5 ± 8.9	3.6 ± 4.1	20.7 ± 11.7
	VF	7.1 ± 0.7	3.1 ± 0.2	3.1 ± 1.5	12.5 ± 0.9	3.9 ± 4.0	13.8 ± 9.0

V, Viruses+Bacteria treatments; VFA, Viruses+Bacteria+Flagellates+Autotrophs treatments; VF, Viruses+Bacteria+Flagellates treatments. LA1, LA2, LB1, LB2: abbreviations as in Table 1.

### Effect of treatments on viral abundance and production

**Figure 2:**
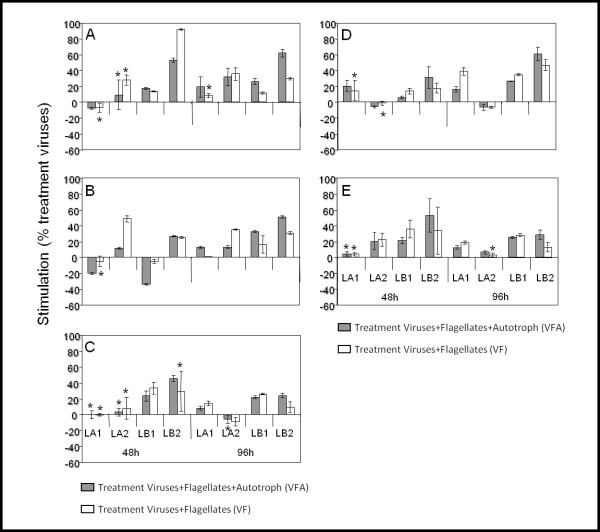
**Stimulation of bacterial abundance (A), production (B) and lysis mortality (C), and viral abundance (D) and production (E)**. Values are given as mean ± standard deviation of triplicats incubations from VF and VFA treatments and average values from the V treatment. ANOVA were preformed between treatment V and the two other treatments (VF and VFA), and the comparison without significance (P > 0.05) was indicated with asterisks.

Viral abundance only varied by a small degree (between 2.9 × 10^7 ^and 4.6 × 10^7 ^virus ml^-1^) in Lake Annecy, while it varied greatly in Lake Bourget particularly during the LB2 experiment (Figure 1). In both LA1 and LA2 experiments, the temporal trend of viral abundance revealed different patterns according to the treatment: viral abundance increased in VF and V treatment, while in the VFA treatment no significant evolution (ANOVA, P > 0.05, n = 9) was recorded (Figure 1). In Lake Bourget, viral abundance increased during the four days of incubation in all treatments, except in treatment V of the LB1 experiment. At the end of incubation, the increase in viral abundance in VF and VFA was significantly higher than in treatment V (ANOVA, P < 0.01, n = 9) in LA1 (+39% and +16%, respectively), LB1 (+34% and +27%, respectively) and LB2 (+47% and +61%, respectively) (Figure 2D). However, the opposite was true for LA2 (-6%, ANOVA, P < 0.05, n = 9). The stimulation of viral abundance was 3-fold higher in Lake Bourget (average +29%) than in Lake Annecy (average +8%) (t test, n = 24, P < 0.001) and significantly different between seasons for each lake (t test, n = 12, p < 0.01).

**Figure 3:**
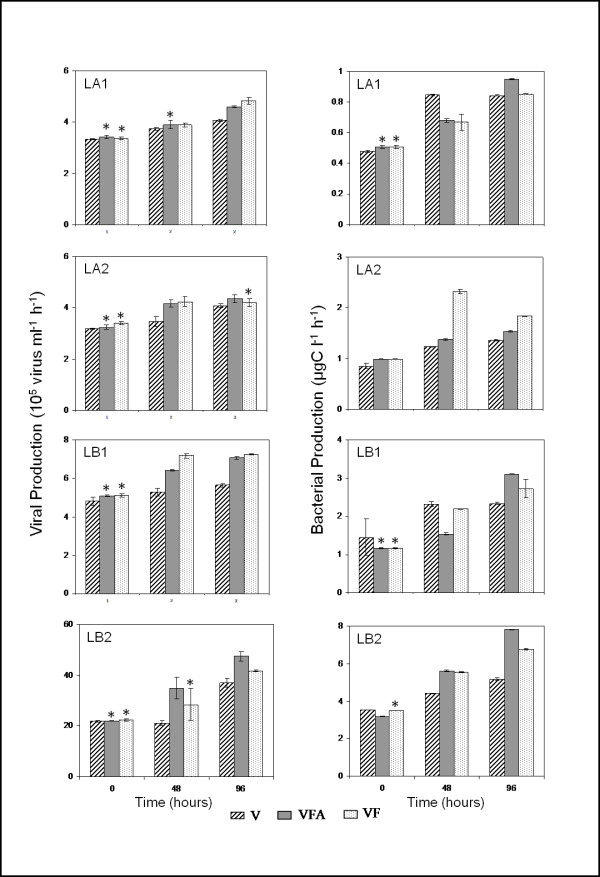
**Time-course of viral production (10^5 ^virus ml^-1 ^h^-1^) and bacterial production (μgC l^-1 ^h^-1^) in the four experiments during the incubation period**. Values are given as mean ± standard deviation of triplicate incubations. Asterisks indicate sampling time point for which VFA and VF treatments were not significantly different from the V treatment (P > 0.05, n = 9, ANOVA). Note that the panels have different scales. LA1, LA2, LB1, LB2: abbreviations as in Table 1.

Similarly to viral abundance, viral production increased without exception from the beginning to the end of the experiments, particularly in VFA and VF treatments whatever the lake (ANOVA, P < 0.05, n = 9). Viral production varied between a minimum of 3.2 × 10^5 ^virus ml^-1 ^h^-1 ^(LA2) and a maximum of 4.7 × 10^6 ^virus ml^-1 ^h^-1 ^(LB2) (Figure 3), which corresponded to 3.5 × 10^5 ^and 47.4 × 10^5 ^cells lysed ml^-1 ^d^-1^, respectively (Table 3). Viral production in VFA and VF were, in most cases, significantly different (ANOVA, P < 0.001, n = 18), over the course of the incubation, being on average 21% higher (range 7-53%) than in V treatments in both lakes (Figure 2E). Stimulation of viral production seemed to be significantly higher (t test, P < 0.0001, n = 24) in Lake Bourget (average +30%) than in Lake Annecy (average +11%), while no significant seasonal differences (t test, P > 0.05, n = 12) were recorded for either lake.

**Table 3:** Bacterial growth rate (r), loss rate, virus-induced mortality and lysis activity rates after 48 h and 96 h

Experiment/Treatment	**Growth rate (r) (d**^**-1**^**)**		**Loss rate of bacteria (d**^**-1**^**)**				**Lysis mortality (d**^**-1**^**)**		**Lysis rate activity (10**^**5 **^**cell ml**^**-1 **^**d**^**-1**^**)**	
	
	48 h	96 h	48 h		96 h		48 h	96 h	48 h	96 h
LA1										
VFA	0.12 ± 0.05	0.14 ± 0.01	-0.03 ± 0.09		-0.06 ± 0.04		0.18 ± 0.01	0.21 ± 0.01	3.90 ± 0.16	4.60 ± 0.04
VF	0.09 ± 0.06	0.10 ± 0.06	0.01 ± 0.04		-0.02 ± 0.01		0.18 ± 0.01	0.22 ± 0.01	3.80 ± 0.07	4.80 ± 0.12
V	0.09 ± 0.06	0.08 ± 0.05					0.18 ± 0.01	0.19 ± 0.01	3.70 ± 0.05	4.10 ± 0.04
										
LA2										
VFA	0.30 ± 0.10	0.37 ± 0.03	-0.02 ± 0.15		-0.08 ± 0.05	*	0.39 ± 0.01	0.41 ± 0.02	4.20 ± 0.14	4.40 ± 0.14
VF	0.36 ± 0.36	0.39 ± 0.01	-0.08 ± 0.05		-0.09 ± 0.05	*	0.40 ± 0.05	0.40 ± 0.02	4.20 ± 0.49	4.20 ± 0.14
V	0.28 ± 0.03	0.47 ± 0.04					0.37 ± 0.02	0.44 ± 0.01	3.50 ± 0.20	4.10 ± 0.07
										
LB1										
VFA	0.27 ± 0.02	0.28 ± 0.01	-0.09 ± 0.01	**	-0.10 ± 0.02	**	0.20 ± 0.01	0.21 ± 0.01	6.40 ± 0.05	7.10 ± 0.09
VF	0.27 ± 0.00	0.23 ± 0.00	-0.05 ± 0.01	**	-0.05 ± 0.01	**	0.22 ± 0.01	0.22 ± 0.01	7.20 ± 0.11	7.20 ± 0.03
V	0.18 ± 0.01	0.18 ± 0.01					0.16 ± 0.01	0.18 ± 0.01	5.30 ± 0.20	5.60 ± 0.08
										
LB2										
VFA	0.68 ± 0.10	0.73 ± 0.01	-0.21 ± 0.01	*	-0.22 ± 0.02	**	1.62 ± 0.19	2.20 ± 0.08	34.9 ± 4.30	47.4 ± 1.83
VF	0.65 ± 0.02	0.62 ± 0.01	-0.18 ± 0.12	*	-0.11 ± 0.01	**	1.32 ± 0.31	1.94 ± 0.03	28.4 ± 6.40	41.7 ± 0.26
V	0.47 ± 0.10	0.51 ± 0.01					1.01 ± 0.04	1.77 ± 0.09	21.1 ± 0.96	36.8 ± 1.75

The significant difference between bacterial growth rate in V treatment and VFA/VF treatments was tested using ANOVA. *, P < 0.05; **, P < 0.001.

### Effects of treatments on bacterial abundance, production and mortality

Bacterial abundance increased throughout the experiments, particularly during the LB2 experiment (Figure 1). Concentrations were significantly higher in VFA and VF than in treatment V (ANOVA, P < 0.05, n = 18). Concentrations in VFA and VF were in most cases similar in Lake Annecy, when compared to each other (ANOVA, P > 0.05, n = 18), in contrast to the significant differences observed in the samples issued from Lake Bourget, with higher bacterial abundance in treatment VFA than VF. At the end of the incubation, the increase in bacterial abundance (comparison of treatments V and both VF and VFA between day 0 and day 4) in treatment VFA was significantly higher than in treatment V (ANOVA, P < 0.01, n = 9) (Figure 2A). In the four experiments, bacterial abundance was significantly higher (by up to 9% to 53%) (t test, P < 0.05) in treatment VFA than in V. In the VF treatment, bacterial abundance was significantly higher (t test, P < 0.05) in LA2 (up to 35%), LB1 (up to 30%) and LB2 (up to 19%) than in treatment V. No significant difference was observed in LA1 (t test, P>0.8). Stimulation of bacterial abundance was significantly different between lakes (t test, P < 0.001, n = 24) (+38% in Lake Bourget and +14% in Lake Annecy) and between seasons with highest values measured in summer (+59% in Lake Bourget and +26% in Lake Annecy).

During the incubation period, bacterial production fluctuated between 0.5 and 0.9 μgC l^-1 ^h^-1 ^in LA1, 0.8 and 2.3 μgC l^-1 ^h^-1 ^in LA2, 1.2 and 3.1 μgC l^-1 ^h^-1 ^in LB1 and between 3.2 and 7.8 μgC l^-1 ^h^-1 ^in LB2 (Figure 3). Following bacterial abundance evolution, a significant increase in the bacterial production (ANOVA, P > 0.05, n = 27) was also recorded throughout the period of incubation. For both lakes, bacterial production was often higher in treatment V than in both VFA and VF during the early spring experiments (LA1 and LB1). After 96 h of incubation, the stimulation of bacterial production (comparison of variation of the viruses treatment (V) and the grazers treatments (VFA and VF)) was observed in all experiments and averaged 27% in treatment VFA and 20.8% in treatment VF when compared to V (Figure 2B). The highest stimulation was observed in VFA during the LB2 experiment (51%). Overall, the bacterial production was significantly different (ANOVA, P < 0.001, n = 27) between the three treatments for the four experiments, with the highest values observed in most cases in VFA and VF (Figures 2 and 3). In contrast to the bacterial abundance, a significant difference in the stimulation of bacterial production was only noted between seasons (t test, P < 0.001, n = 12), with the highest values for summer experiments (+33.5% and +37.5% for Lake Bourget and Lake Annecy, respectively).

Bacterial growth rate fluctuated between 0.1 and 0.7 d^-1 ^after either 48 h or 96 h of incubation (Table 3), with the lowest values recorded during early spring experiments (LA1 and LB1). The presence of flagellates did not induce a reduction of bacterial abundance and the estimation of bacterial loss rates over time generally led to negative values, showing enhanced bacterial growth. In Lake Annecy, this positive impact on bacterial growth was only significant in the LA2 experiment (ANOVA, P < 0.05, n = 6), and was observed in both VF (-0.1 d^-1^) and VFA (-0.1 d^-1^). In Lake Bourget, the two experiments (LB1 and LB2) showed the same effect on bacterial growth, with the highest values observed in VFA treatment (-0.2 d^-1^, ANOVA, P < 0.001, n = 6).

Bacterial mortality due to viral lysis activity was estimated to range between 0.2 d^-1 ^and 2.2 d^-1 ^(Table 3) with the highest values obtained during summer experiments (LA2 and LB2). Differences between V and VFA/VF treatments indicated a significant increase in the lysis mortality rate after 48 h incubation in both LB1 (+28%) and LB2 (+43%) and this enhancement was maintained until the end (96 h) (Figure 2C). In LA1 and LA2, a significant difference between V and the other treatments was observed at the end of incubation, accompanied with an increase in lysis mortality rate in LA1 (+11%), and a decrease in LA2 (-7%).

### Effects of treatments on the bacterial community structure

**Figure 4:**
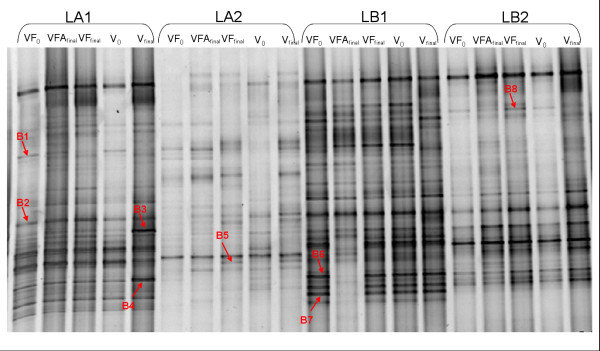
**Bacterial community structure at the beginning (referred to as '0') and at the end (96 h, referred as 'final') of the incubation, visualized by DGGE of PCR-amplified partial 16S rRNA genes, and the position of the different bands excised and sequenced**. (B1 to B8, see Table 5). V_0 _and V_Final_, treatment Viruses+Bacteria at the beginning and the end of experiments; VF_0 _and VF_final_, treatment Viruses+Bacteria+Flagellates at the beginning and the end of experiments, VFA_final_, treatment Viruses+Bacteria+Flagellates+Autotrophs at the end of experiments

Figure 4 shows the PCR-DGGE patterns of the bacterial community structure at the start and end of incubation for the three treatments and the four experiments. Between 17 and 26 bands were found in treatment V, between 18 and 28 in VF and between 18 and 27 in VFA (Figure 4 and Table 4). The number of common bands found in the three treatments for each experiment represented between 24 and 49% (average 40.5%, Table 4). Between 0 and 3 bands (average 3.8%) per experiment were specific to V. Between 0 and 2 bands (average 2.3%) and between 1 and 4 (average 6.5%) bands were specific to VF and VFA, respectively (Table 4).

**Table 4:** Number and specificity of bacterial DGGE bands in the four experiments

	LA1	LA2	LB1	LB2
Total Bands	37	31	34	31
Treatment Viruses	22-23	17-20	26	17-25
Treatment Viruses + Flagellates	25-28	18-19	27	19-23
Treatment Viruses + Flagellates+Autotrophs	25-28	18	24-27	19-27
Bands common in all treatments	47% (16)	42% (13)	49% (18)	24% (13)
Bands specific to:				
Treatment Viruses	0	3% (1)	9% (3)	3% (1)
Treatment Viruses + Flagellates	0	6% (2)	3% (1)	0
Treatment Viruses + Flagellates + Autotrophs	11% (4)	6% (2)	6% (2)	3% (1)
Two combined treatments (VFA + VF)	8% (3)	6% (2)	15% (5)	23% (7)

Among the eight sequenced bands, B2 (excised from the LA1 experiment) was present in all treatments. From this band, ten sequences out of 12 obtained were related to the genus *Curvibacter *(class of β-proteobacteria), the two other sequences corresponding to the genus *Burkholderidia *(class of β-proteobacteria) (Table 5). Three other sequenced bands were visible in all treatments but they increased significantly in intensity at the end of incubation (both B3 and B4 in V_final _of LA1, B8 in VF_final _of LB2). These three excised bands were related to the phylum Actinobacteria (with B3 affiliated to the clade acI) (Figure 4 and Table 5). Finally, the three last bands chosen to be sequenced appeared (B5 in V_final _and VF_final _of LA2) or disappeared (both B6 and B7 in VFA_final _of LB1) at the end of incubation (Figure 4). These ones were all affiliated to the phylum Actinobacteria (as were 85% of the sequenced DGGE bands). Note that the excised band B1 (LA1 experiment), related to the phylum Cyanobacteria (Table 5), disappeared at the end of the incubation in both VF and V treatments.

**Table 5:** Phylogenetic information about the OTUs corresponding to the excised and sequenced DGGE bands

Bands N°	Number of sequenced clones	OTUs	Nearest uncultivated species accession no°,% similarity
B1	12	Phylum: Picocyanobacteria *Synechococcus sp*	AY224199, 98%
B2	10	Class: β-proteobacteria Genus: Curvibacter	EU703347, 98 EU642369, 99%
B2	1	Class: β-proteobacteria Genus: Burkholderia	EU642141, 98%
B2	1	Class: β-proteobacteria Genus: Burkholderia	EU801155, 97% EU63973669, 96%
B3	9	Phylum: Actinobacteria Clade: acI	FJ916243, 99%
B4	11	Phylum: Actinobacteria Unidentified	FN668296, 99%
B5	10	Phylum: Actinobacteria Unidentified	FN668268, 100%
B5	1	Unclassified bacteria	
B6	12	Phylum: Actinobacteria Unidentified	FJ916291, 99%
B7	11	Phylum: Actinobacteria Unidentified	DQ316369, 99%
B8	8	Phylum: Actinobacteria Unidentified	AJ575506, 99%
B8	3	Unclassified bacteria	

**Figure 5:**
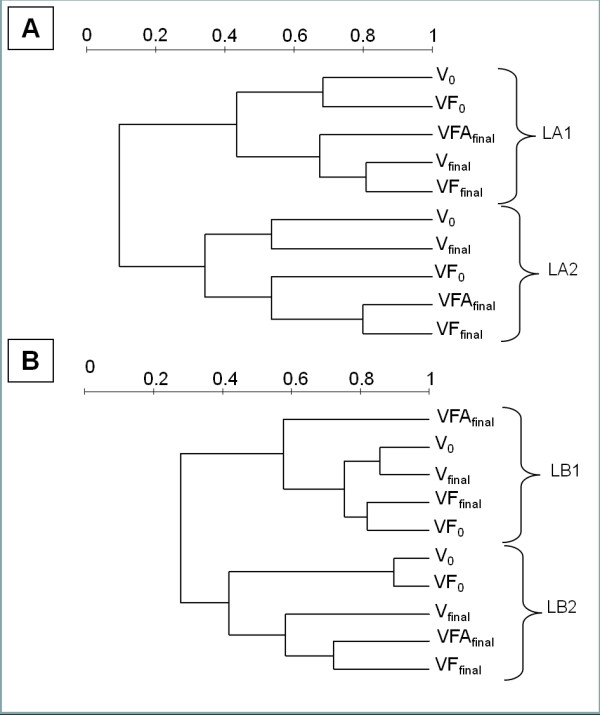
**Cluster analysis of DGGE profiles based on band position and intensity. Scale bars indicated the Bray-Curtis similarity index in Lake Annecy (A) and Lake Bourget (B)**. V_0 _and V_Final_, treatment Viruses+Bacteria at the beginning and the end of experiments; VF_0 _and VF_final_, treatment Viruses+Bacteria+Flagellates at the beginning and the end of experiments, VFA_final_, treatment Viruses+Bacteria+Flagellates+Autotrophs at the end of experiments.

Cluster analyses based on quantification of the band position and intensity (Figure 5) showed that, for each lake, the bacterial community structure was clearly different according to the period (early spring/summer) (Figure 5). In lake Annecy, different responses could be observed following our manipulations: In spring, the effect of time (separation between t0 and tf, whatever the planktonic size fraction) clearly affected the bacterial structure. In summer, the differences in the structure induced by the size fractionation were the strongest, and sample discrimination was clearly linked to the fractionation (1.6 *vs. *5 μm). Similar patterns were obtained for Lake Bourget in summer. Finally, treatment VFA was highly divergent from V and VF (between 42% and 58% of similarity) during the early spring experiment for Lake Bourget.

## Discussion

### Experimental approach

In order to study the influence of both predation pressure and the autotrophic activity on bacterial community of Lakes Annecy and Bourget, we carried out a fractioning approach and performed incubation in either darkness or ambient light. The originality and strength of this study comes from the fact that such experiments were conducted (i) in two ecosystems with either oligotrophic or mesotrophic status and (ii) at two distinct periods of the year (i.e. early-spring and summer) where microbial planktonic dynamics and composition are likely to display clear differences [8,24,25]. Although the use of microcosms may introduce some bias into the development of microbial communities compared with those occurring naturally in the field (due to confinement and handling effects), these experimental tools are still very useful for investigating how processes such as mortality factors induce temporal variation in bacterial dynamics, structure and activity [26]. Incubation time (4 days) coupled with the volume of microcosms (2.5 L) considered in this study have previously been used successfully in other experimental studies [18,22]. We assumed that our design was thus realistic enough compared to the generation time of microorganisms and aimed to obtain significant changes in bacterial and viral activity [27]. A comparison of virus and flagellate abundances at the onset of the experiments with *in situ *conditions and among treatments with different viral and flagellate effects was successful. However, the experimental protocol resulted in a reduction of HNF at the start of the experiment and we thus might have underestimated their influence. Clear effects of HNF were observed at the end of the experiment, when flagellate abundance was about twice as high as *in situ *(Tables 1 and 2).

### Grazing effect on viral activity

According to the model of Miki and Yamamura [28], grazers should reduce the role of the viral loop. As viral production was higher in VF and VFA treatments than in V in most cases, particularly after 48 h incubation, our results instead suggested a stimulation of viral activity in the presence of grazers. Such a stimulation of viral production by the presence of small eukaryotes (grazers) was observed in all experiments for the two lakes. These results corroborate the findings of Jacquet *et al*. [27] who observed a clear and positive relationship between flagellate concentration and VIBM (virus-induced bacterial mortality) in Lake Bourget (r = 0.99, P < 0.05) at three different periods of the year (winter, spring and summer), suggesting a synergistic cooperation between grazer and virus activity. Our new results extend the occurrence of this process at other periods of the year and in the oligotrophic Lake Annecy. Similar beneficial effects of protozoan grazing on viruses have been reported in various lacustrine systems with different trophic statuses [21,23,26]. This means that the trophic status cannot be 'used' as an environmental factor to change the balance between positive and negative effects of flagellates on viruses [29], and it is likely that there are probably different processes involved in enhancing viral activities in response to grazing activity [21].

To the best of our knowledge, Šimek *et al*. [19] were first to suggest that protozoan grazing may influence and increase viral lysis. From that time, other studies reported such a synergistic effect in contrast to freshwater systems [21,26,27]. Nevertheless, an antagonistic interaction between these two compartments was also noted elsewhere [30,31]. Mechanisms by which HNF affect viral activity are still unclear and many hypotheses have been proposed to explain such a cooperative interaction (reviewed by Miki and Jacquet [29]). In brief, grazing activity could stimulate bacterial growth rates, by releasing organic and inorganic nutrients. Higher bacterial growth rates might be associated with enhanced receptor formation on cell surface which may result in a greater chance of phage attachment and *in fine *higher infection frequencies. Thus, grazer stimulation of viral proliferation could occur through cascading effects from grazer-mediated resource enrichment [23]. We observed, in this study, a strong stimulation of bacterial production in treatments with grazers which may corroborate this assumption in both lakes. A link between infection and host production has been reported previously (summarized in Weinbauer [11]) and, recently, experimental studies showed that viruses may preferentially lyse active cells [18,32]. Our results showed that autotrophic activity contributed to this stimulation, mainly in the early summer experiment (for both lakes), while heterotrophic flagellates were always involved in this positive feedback. A shift in the bacterial community structure could also contribute to the synergistic interaction observed in this study. According to Weinbauer *et al*. [33], grazing can favour species which are capable of rapid growth and resistant to grazing, which may stimulate viral infection. Our results also seemed to support this hypothesis since both high bacterial production and specific bands were only observed in treatments VF and VFA.

Stimulation of viral production was much more variable between lakes than between seasons and it was clearly higher in Lake Bourget. This suggests that environmental conditions encountered in the mesotrophic system might promote higher viral activity compared to more oligotrophic conditions. This hypothesis agrees with Lymer *et al*. [34] or Pradeep and Sime-Ngando [26] who observed, during a microcosm experiment, an enhancement of both viral abundance and FIC (frequency of infected cells) in P-enriched samples as a result of nutrient stimulation of bacterial growth, which in turn enhanced viral activity. However, it is noteworthy here that although phosphorus concentration was 2-fold higher in Lake Bourget than in Lake Annecy (Table 1), no significant difference was recorded in bacterial production between the two lakes (t test, P > 0.005). Some studies have suggested that nutrient availability may have an important influence on viral life strategies (e.g. [35,36]). As lysogenic infection is considered the most favourable method of bacterial infection in water characterized by low bacterial abundance and primary production, this may also explain the relatively weak stimulation of viral production observed in Lake Annecy compared to Lake Bourget [32].

In Lake Annecy, and in contrast to viral production, the effects of flagellate presence on viral abundance seemed to be highly variable between the two periods (LA1 *vs. *LA2). This variation revealed viral abundance stimulation in early-spring (LA1) and repression in summer (LA2), for both treatments (VFA and VF). This result could suggest a direct grazing of flagellates on viruses during summer. Virivory by flagellates has been previously reported [37,38] and according to Domaizon *et al*. [39], all flagellates do not act similarly because of large differences between taxon-specifc ingestion rates. During our study, heterotrophic flagellates were mainly represented by *Oikomonas *(45 and 48% during LA1 and LA2, respectively). Also, the grazing impact of flagellates on viruses has always been reported to be relatively low, resulting in < 4% loss [37,38]. Hence, direct grazing of flagellates on viruses was unlikely to explain the repression of viral abundance in LA2. Other factors should be invoked [36] and would need further investigation.

### Effect of both flagellates and viruses on bacterial activity

Higher bacterial production in both VF and VFA treatments than V suggested that grazers and viruses acted additively to sustain (directly or indirectly) bacterial activity in Lake Annecy and Lake Bourget. Such bacterial growth and activity stimulation in the presence of grazers and viruses has been previously reported in the oligotrophic Sep reservoir, Massif Central, France [23,26]. However, the control and reduction of bacterial production by the two mortality agents have been observed in other aquatic systems [18,21,22]. Such variability in possible responses could be due to the initial bacterial community composition and environmental conditions.

The increase in bacterial production with the presence of both predators (flagellates and viruses) could be explained by the fact that grazing activity and viral lysis are likely to release inorganic and organic nutrients which may stimulate bacterial activity. Obviously, the absence of direct measurements of grazing rates of flagellates on heterotrophic bacteria communities, for instance using fluorescently labelled bacteria (FLB) [40], prevented us from drawing firm conclusions about the grazing pressure of HNF on bacteria and our results should be considered in light of that. However, it has been suggested that a minimal proportion of 1,000 heterotrophic bacteria for one heterotrophic flagellate is characteristic of microbial food webs in which flagellates preferentially consume bacteria [39,41,42]. The value for this ratio was higher than 1,000 in each treatment (VFA *vs. *VF) and for each experiment (early spring *vs. *summer). Indeed it varied between 1,632 and 3,866 bacteria per flagellate in Lake Annecy (mean value: 2,795), and between 2,619 and 8,857 in Lake Bourget (mean value: 5899), suggesting that heterotrophic bacteria were abundant enough to support the development of the heterotrophic flagellates that were present.

Seasonal variability in the stimulation of bacterial production seemed to be more important than the trophic status variability, with highest mean values recorded in summer (+33.5% and +37.5% in Lakes Bourget and Annecy, respectively), a period which corresponds to low total phosphorus conditions and high temperature in surface waters (Table 1). Thus, the input of nutrient resources by viral and grazing activities, under such summer conditions, is likely to stimulate the bacterial community much more than under the cold early-spring conditions (temperature = 6-7°C). Moreover, Thomas *et al*. [32] observed that the abundance of HDNA (high nucleic acid containing bacteria) is lower in spring than in summer in Lake Bourget (less than 40% of the total bacterial abundance), and this group is considered to be more active in comparison to LDNA (low nucleic acid bacteria) [43,44]. This could also explain the low stimulation of bacterial production in early spring compared to that in summer.

For most experiments (LA1, LB1 and LB2), the stimulation of bacterial production, at the end of experiments, was much higher in VFA than in the VF treatment (Figure 4) which could be attributed to an increase in substrate availability and regenerated nutrients, resulting from grazing pressure of flagellates on both heterotrophic bacteria and autotrophic communities, in treatment VFA [45,46]. Nevertheless, the opposite situation was observed in Lake Annecy during the summer period (LA2 experiment) suggesting weaker competition by the bacterial community for nutrient resources in the presence of autotrophs, at this period. As a possible explanation, the abundance of autotrophs (represented mainly by picocyanobacteria and PNF) was indeed 2- to 4-fold higher in summer than in early spring while bacterial abundance was 2-fold lower (Table 1).

### Impact of HNF on bacterial community structure

We are aware that the DGGE fingerprinting method presents some bias and only reflects the microorganism populations that are present at relatively high concentrations. For example, while Muyzer *et al*. [47] claimed that the reported sensitivity of DGGE is 1% of the template DNA, Casamayor *et al*. [48] reported that the number of bands is related to the number of populations that account for more than 0.3-0.4% of the total cell counts. In addition, some other bias such as insufficient or preferential disruption of cells during the DNA extraction step, amplification bias (chimera and heteroduplex formation) and band co-migration in the DGGE gel can occur and consequently over- or underestimate the number of bands. However, such limitations are not specific to DGGE and may also be found in other molecular fingerprinting techniques [49]. Therefore, it must be kept in mind that only major changes in the bacterial community composition could be monitored using DGGE. That is exactly what we observed in this study as all sequenced bands belonged to Actinobacteria and Proteobacteria, known to be the most dominant phyla in lakes [50,51]. Thus our results have to be interpreted with caution because the structure of some "non-dominant" phyla, non-detectable with the DGGE technique, could have changed according to the treatments performed in this study.

We found that some bands were specific to each treatment suggesting that some bacterial phylotypes were able to develop and thwart the predation pressure. Such specificity has already been reported in other experimental studies [18,21,22]. Phylotypes, observed in both VFA and VF treatments, were likely to be resistant to both grazing and infection [21,22]. Nevertheless, the presence of phylotypes only in VF (not in VFA) might indicate sensitivity to the autotrophic activity as a result of a weak ability to compete for resources. Phylotypes only present when viruses were the exclusive mortality agents would probably not be able to deal with the combined pressure of grazing and viral lysis [21] or were strongly susceptible to grazing as already suggested by Zhang *et al*. [22]. Finally, the appearance of bands in both VF and VFA treatments could be due to phylotypes benefiting from the presence of predators, e.g., *via *the production of DOM or by the removal of competitors.

A few previous experimental studies have shown that viruses can influence the presence or absence of specific bacterial phylotypes but, typically, result in a reduction of the number of detected phylotypes [53,54]. In contrast, we observed during the summer period an increase in the apparent richness when viruses were the exclusive mortality agents (i.e. the number of detectable bands) giving support to the "killing the winner hypothesis". The stimulation of bacterial diversity in the presence of viruses was also reported in other lacustrine systems by Weinbauer *et al*. [21] and other experimental studies performed in coastal marine systems observed the same trend [18,22]. However, the relative stability of the apparent richness during early spring experiments, in treatment V, highlighted the seasonal variability of virus effects on bacterial diversity. This high variable impact of viruses upon bacterial community structure, already reported by Hewson and Fuhrman [54], could suggest the influence of stochastic processes.

Since no decrease in the number of bands was observed in either treatment VF or VFA, our result could not support the hypothesis of Miki and Yamamura [28] according to whom grazing on infected cells "Kills the killer of the winner" and thus reduces bacterial species richness. In some cases, the combined effect of viruses and flagellates on bacterial fingerprint diversity was more consistent than the effect of viruses alone, suggesting that both predators acted additively to sustain apparent richness. According to Zhang *et al. *[22] the 'killing the winner' hypothesis is mediated by both predators and not just by one type of predator (viruses). Thus, all predators (viruses and flagellates) could act additively in controlling the winners of the competition for resources and caused an increase in detectable phylotypes. In addition, stimulation of bacterial production and related viral lysis also suggested input of nutrients and substrates from grazing and lysis activities which may decrease the competition pressure within bacterial community, thereby increasing the competitiveness of the minor phylotypes [23].

The effect of both predators on the bacterial diversity was not apparent in all experiments, suggesting more variability and complexity in the interactions between bacterial diversity, viruses and grazers than hitherto assumed. Diverse patterns between predators and bacterial diversity were reported in other studies [18,19,55]. Such variability could be explained by the change in the balance between bacterial production and protistan grazing [56] or to chaotic behaviour due to competition among predators for the same prey [28]. Overall, previous work performed in both Lakes Annecy and Bourget, indicated that the strong complexity of the combined physico-chemical and biological parameters (with a larger effect of abiotic factors) is mainly responsible for the evolution of the bacterial community structure [57].

## Conclusion

Many forms of interaction exist between the various components of the microbial loop including the viruses. Our results highlight that viruses and flagellated grazers are likely to act together, synergistically, on bacterial activity and community structure. Even at the community level, interactions between bacterioplankton, viruses and grazers are thus much more complex than hitherto assumed. More than ever, additional studies are needed to fully assess the factors responsible for the variability in the interactions between grazers, bacteria and viruses, especially in freshwater ecosystems, as well as their ecological significance for the microbial community structure/role and whole ecosystem functioning.

## Methods

### Study sites and sampling

Water samples were collected from the two largest natural lakes in France. For the purpose of this study, 40 litres of water samples were collected near the surface (i.e. 2 m) using a water pump and large tubing on 26 March and 10 July 2007 in Lake Annecy (referred to later as LA1 and LA2, respectively) and on 02 April and 17 July 2007 in Lake Bourget (i.e. LB1 and LB2). In this way, for each period, samples were separated by only one week between the two lakes.

### Physicochemical variables

Total organic carbon (TOC) and nutrient concentrations (NH_4_, NO_3_, PO_4_, total phosphorus) were measured at each station and date, according to the standard French protocols AFNOR (details available at http://www.dijon.inra.fr/thonon/les_plateaux_techniques/le_laboratoire_de_physico_chimie). A conductivity-temperature-depth measuring device (CTD SEABIRD SAB 19 Seacat profiler) and a Chlorophyll fluorescence Fluoroprobe (BBE Moaldenke, Germany) were used to obtain vertical profiles of water temperature, conductivity, dissolved oxygen concentration and chlorophyll *a *fluorescence.

### Size fractionation approach

Immediately after sampling, samples were pre-filtered through a 60-μm mesh screen, followed by pre-filtration through Nucleopore membranes (< 5-μm pore size) under low differential pressure (< 50 mm Hg) in order to exclude large eukaryotes. We could thus focus our attention on the small eukaryotes, autotrophic and heterotrophic prokaryotes and viruses. A third of the pre-filtered sample was then filtered through 1.6-μm pore size to yield a total free-living bacteria and 'grazer-free' containing fraction, which was confirmed by detailed microscopic examination at the beginning and at the end of the experiments. The remaining pre-filtred sample was divided into two parts; one of them was kept in a black box (simulating darkness) to inhibit the autotrophic activity. Therefore, three combinations of treatments were performed: the treatment 'Viruses + Bacteria + heterotrophic Flagellates (grazers) + Autotrophs' (fraction < 5 μm, referred to as VFA); the treatment 'Viruses + Bacteria + Flagellates (grazers)' (fraction < 5 μm put into a black box; VF) and finally the treatment 'Viruses + Bacteria', i.e. without the flagellates and the autotrophic community (fraction < 1.6 μm, referred as V).

Samples so transformed were divided into triplicates and poured into 2.5 L Nalgene transparent carboys, which had been previously cleaned with 1.2 N HCl and rinsed three times with Milli-Q and filtered lake water. All the bottles were incubated in the lake at 2 m depth for four complete days. Subsamples were taken from each triplicate at day 0, 2 and 4 to assess microbial abundances and activities, and at day 0 and 4 for the analysis of the bacterial community diversity.

### Flow cytometry (FCM) sample analysis

We used a FACSCalibur flow cytometer (Becton Dickinson, Franklin Lakes, NJ, U.S.A.) equipped with an air-cooled laser providing 15 mW at 488 nm with the standard filter set-up. Only a few hours after sampling (less than 4 h), one millilitre of water was immediately analysed without adding any fixative or dye to analyse the picocyanobacterial community dynamics and also to check for the absence/presence of prokaryotic (e.g. *Synechococcus*) and eukaryotic autotrophic organisms in the V treatment. Such unfixed samples, kept in darkness in refrigerated boxes and at 4°C for a few hours before analysis, revealed no significant changes in cell counts while this was not true when using either formaldehyde or glutaraldehyde (not shown). Fluorescent microbeads (Molecular Probes Inc., Eugene, OR, U.S.A.) of 1-μm diameter were added to each sample as an internal standard. Another 1 ml was fixed and used for bacterial and viral counts via FCM, according to the protocol described in Personnic *et al. *[25]. Briefly, viruses were fixed with glutaraldehyde (0.5% final concentration, grade I, Merck) for 30 min in the dark, then diluted in 0.02 μm filtered TE buffer (0.1 mM Tris-HCL and 1 mM EDTA, pH 8), and incubated with SYBR Green I (at a final 5 × 10^-5 ^dilution of the commercial stock solution; Molecular Probes), for 5 min at ambient temperature, followed by 10 min at 75°C, and then another 5 min at room temperature, prior to FCM analysis. Heterotrophic bacterial counts were performed on samples that had also been fixed with glutaraldehyde (0.5% final concentration) for 30 minutes, but the samples were then diluted in 0.02 μm filtered deep-lake water sample, and incubated with SYBR Green I (10^-4 ^dilution of the commercial stock solution) for 15 min [25] Listmode files were analysed using Cytowin [58].

### Enumeration of flagellates

50 ml sub-samples were fixed with glutaraldehyde (1% final concentration), stained with primuline [59] and collected onto black polycarbonate membranes (0.8-μm pore size). For flagellates, slides were prepared within 24 h after sampling and were stored at -25°C in darkness to minimise the loss of autofluorescence [60]. Slides were observed at a 1,250× magnification using an epifluorescence microscope (Nikon Eclipse TE200) under UV light for heterotrophic nanoflagellates and, under blue and green light for pigmented nanoflagellates.

### Bacterial production

The incorporation of ^3^H-leucine was determined following the protocol of Kirchman [61]. For each sample, 5 sterile eppendorfs (2 ml) received 1 ml of sub-sample. Two samples were fixed with formaldehyde (1.6% final conc.) to serve as controls. Eppendorfs were inoculated with known saturating ^3^H-Leu (80 nM final concentration, specific activity: 73 Ci.mmol^-1^) and incubated in the dark for 2 h. Protein synthesis was stopped by the addition of formaldehyde (1.6% final concentration). Samples were then filtered through a 25-mm diameter, 0.22-μm pore size membrane (GTTP). The filters were then rinsed twice with 5 ml of trichloroacetic acid (TCA, 5% final concentration). The filters were placed in scintillation vials, allowed to dry and solubilised with 1 ml of toluene. After adding 3 ml of the scintillation cocktail (Hionic Fluor, Perkin Elmer), the radioactivity was counted with a Packard Tricarb Liquid Scintillation Analyser 1500. Bacterial production, calculated in pmoles l^-1 ^h^-1 ^of ^3^H-Leucine incorporated into protein, was converted in μgC l^-1 ^h^-1 ^following Simon and Azam [62]: BP (μgC l^-1 ^h^-1^) = Leu (mmols Leu L^-1 ^h^-1^) × 131.2 × (%Leu)^-1 ^× (C:Protein) × ID); with C:protein = 0.86 (ratio of cellular carbon to protein); %Leu = 0.073 (fraction of leucine in protein). ID = 1 (Isotopic Dilution); 131.2 = Molecular weight of the leucine.

### Estimation of viral production

We used the dilution technique of Wilhelm *et al. *[63] in order to estimate the viral production throughout the experiment at day 0, 2 and 4. 50 ml of sub-samples were diluted and mixed with 100 ml of virus-free (0.02-μm pore size pre-filtered at day 0 and kept at 4°C) lake water, and incubated in dark conditions. Triplicates were made and incubated at *in situ *temperature in the dark. One-ml sub-samples were collected at 0, 3, 6, 12, 18 and 24 h. Viral production rates were determined from first-order regressions of viral abundance versus time after correcting for the dilution of the bacterial hosts between the samples and the natural community, a necessary step to account for the loss of potentially infected cells during the filtration. Viral production (VP, virus ml^-1 ^h^-1^) was calculated as proposed by Hewson and Fuhrman [64]: VP = m × (b/B) where m is the slope of the regression line, b the concentration of bacteria after dilution, and B the concentration of bacteria prior to dilution.

We estimated the number of lysed bacteria (cell ml^-1 ^h^-1^) during the viral lysis activity by considering an average burst size (27) previously estimated for Lake Bourget [7,65] with the number of lysed bacteria = Viral production/Burst Size [66].

In order to show the effect of the presence of flagellates on the dynamics and activities of both heterotrophic bacteria and viruses, we calculated the stimulation of the different parameters presented above (both abundance and production) in treatments VF and VFA (as proposed by Bonilla-Findji *et al. *[18] and Zhang *et al. *[22]). The stimulation corresponds to the difference in variation between treatments with flagellates (VFA or VF treatments) and the treatment without flagellates (V treatment) between 0 and 48 h, and between 48 h and 96 h, respectively. As an example, the equation used to calculate the stimulation of bacterial abundance in the VF treatment, between 0 and 48 h, is:(1)

Where BA_(VFA)0 _and BA_(VFA)48 _are the abundance of bacteria in VFA, at the beginning and after 48 h of incubation and BA_(V)0 _and BA_(V)48 _are the abundance of bacteria in V, at the beginning and after 48 h of incubation.

### Net growth and loss rates of bacteria

Bacterial net growth rates with bacterial predators (rb, d^-1^) and without predators (r, d^-1^) were calculated from the difference in abundances from day 0 to day 2 (t = 48 h) and from day 0 and day 4 (t = 96 h), assuming exponential growth. We used the equations: rb = (ln Nbt - ln Nb0)/t and r = (ln Nt - ln N0)/t; where N0 and Nt are the bacterial abundances (Nb0, Nbt = with predators (VFA, VF), N0, Nt = without predators (V)) at the beginning and after 48 h or 96 h of incubation. The loss rate of bacteria due to grazing activities were calculated as the differences between the treatment with (VFA, VF) and without (V) predators: g = r - rb [67].

### Nucleic acid extraction, PCR and DGGE

Analysis of the bacterial community structure was assessed using Denaturing Gradient Gel Electrophoresis (DGGE). Bacteria were harvested from approximately 250 ml water onto 47-mm diameter, 0.2-μm pore size, polycarbonate white membrane filters (Nuclepore) after a pre-filtration step through 2-μm pore size polycarbonate membrane filters (Nuclepore) to eliminate large eukaryotes and filamentous cyanobacteria. The filters were then stored at -80°C prior to nucleic acid extraction. Nucleic acid extraction was performed as described in Dorigo *et al. *[68]. Molecular weight distribution and purity of the DNA were assessed by 1% agarose gel electrophoresis and quantified by both visual comparison with molecular weight markers in ethidium bromide stained agarose gels (rough estimate) and by optical density measurements using NanoDrop ND-1000 Spectrophotometer (Thermo Scientific). Such material was then stored at -20°C until PCR amplification.

PCR reactions were carried out using the Eubacteria-specific primer 358-GC [47] and the universal primer 907 rM [69] which amplify the variable V3 region of the 16S rRNA gene and yield a DNA fragment of ca. 550 bp. All PCR amplifications were carried out using about 30 ng of extracted DNA in a 50 μl reaction mix containing 10 × *Taq *reaction buffer (Eurobio), 1.5 mM MgCl_2_, 120 μM of each deoxynucleotide, 1 μM of each primer, bovine serum albumin (Sigma, 0.5 mg ml^-1 ^final concentration), and 1.25 U *Taq *DNA polymerase (Eurobluetaq, Eurobio). PCR amplification consisted of an initial denaturation step of 94°C for 5 min, followed by 30 cycles of denaturation at 94°C for 1 min, annealing at 52°C for 1 min, and extension at 72°C for 1 min, and a final elongation step at 72°C for 5 min using a PTC100 thermocycler (MJ Research). Correct size (ca. 500 bp length) of PCR products were determined by 2% agarose gel electrophoresis with a DNA size standard (Low DNA Mass Ladder, GIBCO BRL).

DGGE analysis was performed on PCR fragments, as described in Berdjeb *et al*. [57] using *Ingenyphor U-2*^® ^(Ingeny international) and by using a 40-80% gradient. Since all of the replicates (more than 70) could not be placed in the same gel, aliquots of DNA extracts from the three replicates of each treatment were pooled, but only after we had checked similarity in DGGE patterns between replicates for all sampling time points. Digital images of the gels were obtained using a Kodak DC290 camera, and were then saved for further analysis using the Microsoft Photo Editor Software. The DGGE banding patterns were analyzed using the GelCompare II software package (Applied Maths, Kortrijk, Belgium) and after digitalization of the DGGE gels. Briefly, banding patterns were first standardized with a reference pattern included in all gels. Each band was described by its position (Y, in pixel on the image file) and its relative intensity in the profiles (P_i_) which could be described as the ratio between the surface of the peak (n_i_) and the sum of the surfaces for all the peaks within the profile (N).

### Cloning-sequencing

From the DGGE gels, the bands of interest were excised, placed in sterile water and stored at -20°C. Prior to cloning, each excised DGGE band was subjected to a freeze-thaw cycle and then centrifuged. DGGE fragments contained in the supernatant were used as template in a second PCR amplification performed as described above. The resulting PCR products were cloned with an Invitrogen cloning kit (TOPO TA cloning) according to the manufacturer's instructions. Twelve clones were randomly chosen for each band of interest. Each clone was verified by PCR using the commercial primers M13 and finally sequenced (GATC Biotech). Sequences were then edited, aligned with Genedoc [70] and finally checked for chimeras using Bellerophon [71] and the Ribosomal Database Project (RDP) [72]. Sequences were finally subjected to BLAST and the RDP database to determine the level of similarity with other 16S rRNA gene sequences available in Genbanks.

### Statistical Analysis

Differences between treatments per experiment, per time point were tested for significance using parametric analysis of variance (ANOVA) including post hoc test analysis (Fisher's protected least significant difference test). Testing for normality and homogeneity of variance was performed, and data transformation was done when required (for all data compared per test). Differences were considered significant at P value of < 0.05. We compared the difference on the stimulation rate of abundance and production of both viral and bacterial communities according to the seasons (n = 12) and trophic status (n = 24) by using paired t test.

## Authors' information

LB and TP have been PhD students, working in the BioFEEL group between 2007 and early 2011. ID and SJ have obtained permanent positions since 2000, as research scientists.

## Authors' contributions

All authors read and approved the final manuscript. SJ was the responsible of this study and participated in the experimental design. LB realised all analyses except for the flagellate counting and phylotype analysis. TP made the cloning-sequencing analysis of the selected DGGE bands. ID participated to the experimental design and realised the flagellate counting. Writing was mainly done by LB, helped and corrected by ID and SJ.
